# Implementation of latent tuberculosis infection screening and treatment among newly arriving immigrants in the Netherlands: A mixed methods pilot evaluation

**DOI:** 10.1371/journal.pone.0219252

**Published:** 2019-07-01

**Authors:** Ineke Spruijt, Connie Erkens, Jeanine Suurmond, Erik Huisman, Marga Koenders, Peter Kouw, Sophie Toumanian, Frank Cobelens, Susan van den Hof

**Affiliations:** 1 KNCV Tuberculosis Foundation, The Hague, The Netherlands; 2 Department of Public Health, Amsterdam Public Health Research Institute, Amsterdam University Medical Center, University of Amsterdam, Amsterdam, The Netherlands; 3 Department tuberculosis control, Public Health Service Haaglanden, The Hague, The Netherlands; 4 Department tuberculosis control, Public Health Service Gelderland Zuid, Nijmegen, The Netherlands; 5 Department tuberculosis control, Public Health Service Amsterdam, Amsterdam, The Netherlands; 6 Department tuberculosis control, Public Health Service Twente, Enschede, The Netherlands; 7 Department of Global Health and Amsterdam Institute for Global Health and Development, Amsterdam University Medical Center, Amsterdam, The Netherlands; Agencia de Salut Publica de Barcelona, SPAIN

## Abstract

**Introduction:**

To reach pre-elimination levels of tuberculosis (TB) incidence in the Netherlands, prevention of TB among immigrants through diagnosis and treatment of latent TB infection (LTBI) is needed. We studied the feasibility of a LTBI screening and treatment program among newly arriving immigrants for national implementation.

**Methods:**

We used mixed methods to evaluate the implementation of LTBI screening and treatment in five Public Health Services (PHS) among immigrants from countries with a TB incidence >50/100,000 population. We used Poisson regression models with robust variance estimators to assess factors associated with LTBI diagnosis and LTBI treatment initiation and reported reasons for not initiating or completing LTBI treatment. We interviewed five PHS teams using a semi-structured method to identify enhancing and impeding factors for LTBI screening and treatment.

**Results:**

We screened 566 immigrants; 94 (17%) were diagnosed with LTBI, of whom 49 (52%) initiated and 34 (69%) completed LTBI treatment. LTBI diagnosis was associated with male gender, higher age group, higher TB incidence in the country of origin and lower level of education. Treatment initiation was associated with PHS (ranging from 29% to 86%), lower age group, longer intended duration of stay in the Netherlands, and lower level of education. According to TB physicians, clients and their consulted physicians in the home country lacked awareness about benefits of LTBI treatment. Furthermore, TB physicians questioned the individual and public health benefit of clients who return to their country of origin within the foreseeable future.

**Conclusions:**

Doubt of physicians in both host country and country of origin about individual and public health benefits of LTBI screening and treatment of immigrants hampered treatment initiation: the high initiation proportion in one PHS shows that if TB physicians are committed, the LTBI treatment uptake can be higher.

## Introduction

Tailored screening and treatment programs for latent tuberculosis (TB) infection are needed for low TB incidence countries (TB incidence <10/100,000 population) to reach pre-elimination (TB incidence < 1/100,000 population) levels of TB. [1, 2] In the Netherlands (TB incidence of 5.2/100,000 population (2016)), TB incidence is still high among the foreign-born population (TB incidence of 35.3/100,000) who count for 75 percent of all TB cases. [1, 3] New immigrants from non-European Union (EU) countries with a TB incidence >50/100,000 population undergo mandatory TB screening at entry in the Netherlands. Additionally, immigrants from countries with a TB incidence >200/100,000 population are offered voluntary two-year biannual follow-up screening. Current screening policies are however unlikely to effectuate a decline in TB incidence needed to reach pre-elimination.

Currently, screening for TB among immigrants age ≥18 years is done with a chest X-ray (CXR). Since 2016 -concurrent with this study- implementation of LTBI screening and treatment among immigrants age <18 years started. [4–6] The challenge with current CXR follow-up screening among immigrants is that the coverages are low (44% in the first round and 12% in the last round) and only 48% of incident TB cases among immigrants are identified through this screening.[6] Furthermore, 60% of all foreign-born TB patients lived in the Netherlands greater than 2.5 years and were no longer eligible for TB screening. [7] LTBI screening at entry may overcome these challenges, is proven to be cost-effective and would contribute to a decline in TB incidence. [8, 9]

The Netherlands has long-standing experience with management of LTBI among TB contacts and occupational risk groups. [4, 5] However, the impact of tailored approaches for LTBI screening and treatment programs among immigrants are unknown. A feasibility study identified barriers for successful implementation of the intervention in this group: TB staff had limited competencies to educate on LTBI screening and treatment; out of pocket expenditure for treatment and unfamiliarity with LTBI treatment in the home country impeded LTBI treatment initiation, and short duration of stay in the Netherlands jeopardized treatment completion. [10]

In this pilot study, we addressed the identified barriers by developing tailored information materials in Dutch and English. Additionally, we offered free LTBI treatment and we set a minimum intended stay in the Netherlands of six months for immigrants to be screened to ensure continuity of LTBI treatment care. We used a mixed methods study design to address the following research questions: 1) What is the prevalence of LTBI at arrival among immigrants? 2) What is the initiation and completion proportion of LTBI treatment? 3) Has LTBI treatment initiation improved when offering LTBI treatment free of charge? 4) What are reasons for not initiating or completing LTBI treatment? and 5) What remaining enhancers and barriers do TB care staff experience during the LTBI screening and treatment?

## Materials and methods

### Study population and setting

From March 2016 until September 2016, five (20% of a total of 25) Public Health Services (PHS) in urban (n = 2) and rural (n = 3) parts of the Netherlands replaced mandatory CXR entry-screening with screening for both TB disease and LTBI using a health assessment and tuberculin skin testing (TST) with confirmatory Interferon Gamma Release Assay (IGRA) or IGRA alone. Clients with a positive IGRA test result or clients with symptoms suggestive for TB disease, relevant comorbidities or using immunosuppressive medication were additionally screened with CXR and consulted by a TB physician.

We included immigrants, not applying for asylum, from non-EU countries with a TB incidence of >50/100,000 population, of all ages, with an intended stay in the Netherlands of at least six months, and no history of TB. Immigrants who did not meet inclusion criteria were screened according to current policy: LTBI screening for those age <18 years and CXR for those age ≥18 years. A detailed description of current TB screening policies in the Netherlands can be found elsewhere [4, 5, 11].

### LTBI screening and treatment process

We developed a study protocol on which we trained PHS staff. Clients received an information brochure about the purpose of the LTBI screening either by mail or at registration at the PHS. Medical Technical Assistants (MTA), i.e., healthcare assistants trained to perform TB screening activities (symptom screening, tuberculin skin test and chest X-ray), BCG vaccination, to give information on TB screening procedures, to document screening results and to perform specific administrative tasks, handled registration of the client and gave further explanation about the screening procedures. Consequently, clients completed a health questionnaire which was evaluated by MTAs to identify clients with: 1) a history of TB disease or LTBI treatment, 2) symptoms suggestive for TB disease, 3) BCG vaccination or recent (other) vaccinations, and 4) relevant comorbidities or immunosuppressive medication use, which may affect the validity of test results. (S1 Fig)

Two PHSs screened directly with IGRA and three PHSs used TST followed by confirmatory IGRA. Children <12 years with normal immunity were screened with initial TST followed by confirmatory IGRA. PHS used PPD Tuberculin mammalian (BulBio, Sofia, Bulgaria, 0.1 ml intracutaneous) for TSTs. MTAs interpreted TST results after 48–72 hours by measuring the size of induration (mm): indurations between 3 and 10mm were double read by a second MTA. According to the national policy for LTBI testing, TST indurations ≥5mm were confirmed with IGRA: using QuantiFERON-TB Gold Plus (QFT-Plus; Qiagen, Germantown, MD) according to manufacturer’s instructions. [12] The LTBI diagnosis was considered confirmed in those with a QFT-plus test result ≥0.35 IU/ml, after exclusion of TB disease through physical examination, CXR and -if indicated- bacteriological investigation.

TB physicians initiated and monitored LTBI treatment of clients following the national guideline for LTBI treatment. After confirmation of the LTBI diagnosis, TB physicians ruled out contra-indications for LTBI treatment and transaminase levels were assessed in clients with a history of liver disease, alcohol abuse, HIV infection, age >35 years, pregnancy and during the first three months of post-partum. If eligible for LTBI treatment, TB physicians educated the client on the purpose and possible side-effects of LTBI treatment and offered each client LTBI treatment with 3 months rifampicin /isoniazid combination therapy. In accordance with national guidelines, clients with contra-indication or clients who were not motivated to initiate LTBI treatment were offered bi-annual follow-up screening for a period of two years. [13]

TB physicians consulted clients on LTBI treatment every month for adverse events (including hepatotoxicity tests) and treatment adherence. A CXR was repeated after one month and at the end of the treatment to rule out the development of thoracic TB disease. During regular contact moments, TB nurses interviewed, educated and supported the client during the treatment in a demand-driven, tailored way. [13]

### Data collection

#### Quantitative data

We collected the following information from each client: intended length of stay in the Netherlands, reason of stay in the Netherlands, current employment status, and current health insurance status. We retrieved screening results, diagnostic and treatment data from the electronic TB client registration systems used by the PHSs in the Netherlands. For all clients diagnosed with LTBI or TB we retrieved data from the Netherlands TB Register: a database of patients diagnosed with TB disease and LTBI in The Netherlands. To evaluate LTBI treatment, TB physicians or TB nurses filled out a questionnaire about perceived language barriers, reasons for not initiating, discontinuing, or not completing LTBI treatment, the occurrence of side effects, other challenges encountered during the treatment, and the frequency of supervision.

#### Qualitative data

We used semi-structured group interviews to identify enhancers and barriers for LTBI screening and treatment as experienced by PHS staff. We interviewed each of the 5 PHS teams at their department for approximately one hour. In each interview at least one MTA, one TB nurse, and one TB physician participated. We based the topic guide on Lévesque’s conceptual framework for access to care [14] which uses five dimensions in a systematic pathway for access to care: approachability, acceptability, availability and accommodation, affordability, and appropriateness. S1 Table provides an explanation of Lévesque’s Conceptual framework for Access to care and the topic guide.

### Data analyses

We double entered data from questionnaires in MS-Access (Microsoft Corp, Seattle WA, USA) and checked for inconsistencies against the raw data. After merging the datasets, we checked inconsistencies between the three datasets, for which we approached the PHS for validation and substitution of missing data. We calculated proportions for clients’ characteristics, and the cascade of screening and care: LTBI test results, LTBI treatment initiation and completion, including reasons for not initiating or completing the LTBI treatment.

We assessed factors associated with LTBI diagnosis and LTBI treatment initiation using full case analyses: cases with missing values were excluded from the analyses (n = 33 and n = 7 respectively). As probabilities for outcome occurrence (18% and 52% respectively) were not rare, univariable Poisson regression models with robust variance estimators were used to calculate risk ratios. [15] For LTBI diagnosis, we also developed a multivariable Poisson regression model using backward elimination of the initial model with variables yielding a p-value <0.1 in univariable analysis, guided by changes in regression coefficients and changes in the fit, as indicated by -2loglikelihood. We checked for interaction between independent variables before fitting multivariable Poisson regression models. Due to small numbers, we combined categories of variables in regression analyses, we did not perform multivariable analysis for LTBI treatment initiation and we did not calculate risk ratios for LTBI treatment completion. We recoded and analyzed data using SPSS Statistics 25.0 (IBM Analytics, Chicago, IL, USA).

We verbally transcribed all audiotaped interviews. After familiarization with the data, IS developed a coding scheme to guide the coding of all transcripts. We refined the coding scheme along the coding process. In regular meetings, IS and JS discussed coding and interpretation of the data. [16] We used MAXQDA (Version 11, VERBI GmbH, Berlin, Germany) to assist in analyses of qualitative data.

#### Ethics statement

The Medical Ethical Committee (METC) of University Medical Center Amsterdam (UMC-AMC) waived the need for ethical approval of the study because the Dutch Medical Research Involving Human Subjects Act does not apply given that the study was primarily focused on finding and treating TB, and in the Netherlands, Public Health Services are licensed to conduct screening for TB infection.

The qualitative component of this study did not require approval because respondents interviewed were health care providers and not patients. We followed the ethical principles for medical research involving human subjects as laid down in the Declaration of Helsinki and adopted by the World Medical Association (WMA Declaration of Helsinki 2000). Each respondent was adequately informed about the aims and methods of the study and audio-taped verbal a priori informed consent was obtained from the respondents for their participation in this study. Audiotaped verbal consent was sufficient because no personal information has been used and the individual's identity has been protected by removing any personal identifiers from the data. Codes were used to designate the respondents to guarantee their anonymity.

Patients from Public Health Services are routinely informed that anonymized data from their patient records can be used for evaluation purposes. For this study, we obtained pseudonymized data from patient records from the Public Health Services, from the Dutch National Register for Tuberculosis and from the health questionnaires. Pseudonymized data were merged and made fully anonymous by a trusted third party before data analyses.

## Results

**Fig 1:**
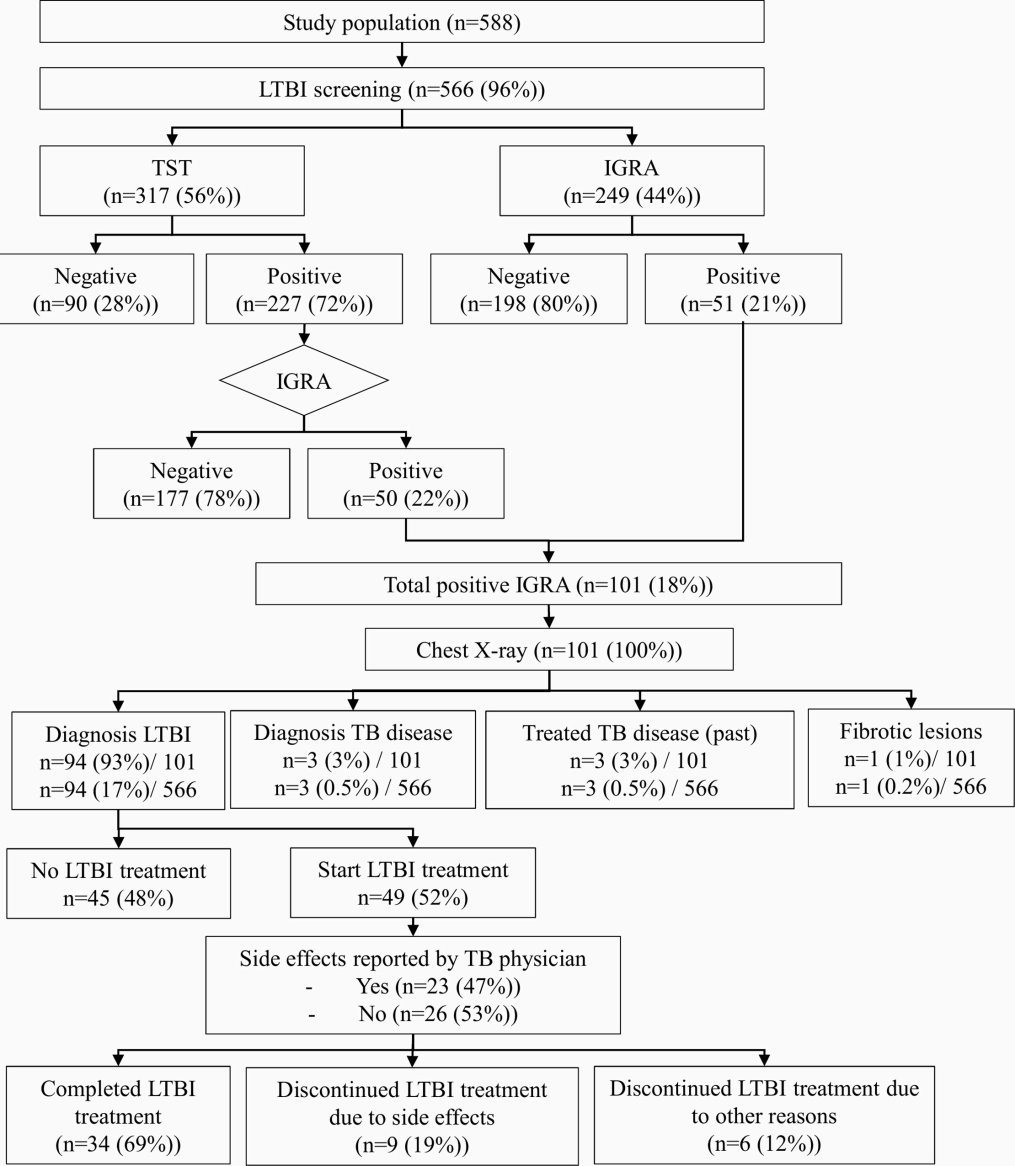
Flowchart of latent tuberculosis infection screening and treatment results.

In total, 588 clients were eligible for LTBI screening, which was completed by 566 (96%). Reasons for not completing LTBI screening were: refusal of LTBI screening (n = 2), failed blood sampling (n = 5), refusal of second blood sampling after failed first IGRA (n = 3), unknown (n = 12). (Fig 1)

### Characteristics of clients screened for LTBI

Of 566 clients screened for LTBI, the majority were female, aged between 18–34 years, had higher levels of education, intended to stay more than one year in the Netherlands, and originated from countries with a TB incidence >200/100,000 population. At the time of the screening, 157 (28%) clients did not have health insurance. (Table 1)

**Table 1:** Descriptive statistics of the study population.

Characteristics	LTBI screening		LTBI diagnosis		Initiate LTBI treatment		Complete LTBI treatment	
	n	Colum (%)	n	Row (%)	n	Row (%)	n	Row (%)
**Total**	**566**	**(96)**	**94**	**(17)**	**49**	**(52)**	**34**	**(69)**
**Public Health Service (PHS)**								
PHS 1	158	(28)	21	(13)	18	(86)	12	(67)
PHS 2	74	(13)	7	(10)	2	(29)	1	(50)
PHS 3	198	(35)	40	(20)	15	(38)	10	(67)
PHS 4	59	(10)	14	(24)	8	(57)	6	(75)
PHS 5	77	(14)	12	(16)	6	(50)	5	(83)
**Gender**								
Female	329	(58)	45	(14)	24	(53)	15	(63)
Male	237	(42)	49	(21)	25	(51)	19	(76)
**Age**								
0–17	85	(15)	4	(5)	3	(75)	3	(100)
18–24	64	(11)	7	(11)	5	(71)	3	(60)
25–34	286	(51)	48	(17)	27	(56)	19	(70)
35–44	101	(18)	24	(24)	11	(46)	6	(55)
≥ 45 years	30	(5)	11	(37)	3	(27)	3	(100)
**TB incidence country of origin**								
50-99/100.000	144	(25)	11	(8)	6	(55)	2	(33)
100-199/100.000	85	(15)	21	(25)	15	(71)	11	(73)
≥200/100.000	337	(60)	62	(18)	28	(45)	21	(75)
*India*	*202*	*(36)*	*36*	*(18)*	*12*	*(33)*	*9*	*(75)*
*Other countries*	*135*	*(24)*	*26*	*(19)*	*16*	*(62)*	*12*	*(75)*
**Top 10 countries of origin**								
India	202	(36)	36	(18)	12	(33)	9	(75)
China	59	(10)	5	(9)	3	(60)	1	(33)
Indonesia	34	(6)	5	(15)	2	(40)	2	(100)
Russia	33	(6)	1	(3)	0	(0)	-	-
Morocco	32	(6)	11	(34)	10	(91)	8	(80)
Philippines	28	(5)	4	(14)	4	(100)	4	(100)
South of Africa	28	(5)	5	(18)	5	(100)	3	(60)
Ukraine	18	(3)	2	(11)	2	(100)	1	(50)
Thailand	18	(3)	2	(11)	0	(0)	-	-
Pakistan	17	(3)	4	(24)	2	(50)	2	(100)
**Intended stay in the Netherlands**								
6 months, < 1 year	59	(10)	10	(17)	4	(40)	3	(75)
1 year, < 5 years	273	(48)	37	(14)	14	(38)	9	(64)
≥ 5 years	225	(40)	45	(20)	31	(69)	22	(71)
Missing	9	(2)	2	(22)	0	(0)	-	-
**Employment status**								
Employed	205	(36)	38	(19)	16	(42)	8	(50)
Unemployed	200	(35)	42	(21)	25	(60)	19	(76)
School	140	(25)	9	(6)	5	(56)	5	(100)
Missing	21	(4)	5	(24)	3	(60)	2	(67)
**Highest completed education**								
Lower / no formal education	38	(7)	12	(32)	11	(92)	7	(64)
Secondary education	51	(9)	10	(20)	7	(70)	5	(71)
Higher education	456	(81)	67	(15)	28	(42)	21	(75)
Missing	21	(4)	5	(24)	3	(60)	1	(33)
**Health insurance** ^**a**^								
No	157	(28)	27	(17)	20	(74)	14	(70)
Yes	376	(66)	61	(16)	27	(44)	18	(67)
Missing	33	(6)	6	(18)	2	(33)	2	(100)
**BCG vaccinated**								
No	146	(26)	24	(16)	15	(63)	8	(53)
Yes	252	(45)	42	(17)	20	(48)	17	(85)
Missing	168	(30)	28	(17)	14	(50)	9	(64)
**Immunocompromised** ^**b**^								
No	558	(99)	92	(17)	48	(52)	33	(69)
Yes	8	(1)	2	(25)	1	(50)	1	(100)

^a^ Health insurance at time of the LTBI screening

^b^ Clients with conditions associated with immunosuppression: inflammatory bowel disease, kidney failure/dialysis, cancer, organ transplantation, psoriasis, rheumatism, sarcoidosis, silicosis, or medications such as: prednisone/dexamethasone/methotrexate, TNF-alpha blockers (biologicals), cancer medication, medication following organ transplantation

### LTBI screening and treatment results

Of 317 clients tested with TST, 227 (72%) had a positive TST, of whom 50 (22%) had a positive IGRA. Of 249 clients screened directly with IGRA, 51 (20%) tested positive. In total 101 (18%) clients had a positive IGRA, of whom 94 (17%) were diagnosed with LTBI, three clients (0.5%) were diagnosed with extrapulmonary TB disease, three clients (0.5%) had (treated) TB disease in the past, and one (0.2%) client had fibrotic lesions of presumed tuberculous origin. (Fig 1)

The risk of being diagnosed with LTBI was higher for men (1.5 times; 95% CI 1.0–2.2) than for women; higher for clients aged 25–34 years (2.5 times; 95%CI: 1.3–4.8) or 35 years and older (3.5 times; 95%CI: 1.8–6.8) than for clients aged 0-24-year; higher for clients from countries with a TB incidence >100/100,000 population than for those from countries with lower TB incidence; and higher for clients with lower levels of education (1.6 times; 95% CI 0.9–2.6) than for clients with higher levels of education. (Table 2)

**Table 2:** Results of Poisson regression model with robust variance estimators.

	LTBI diagnosis ^a^						LTBI treatment initiation ^a^			
	Descriptive		Unadjusted RR^b^		Adjusted RR		Descriptive		Unadjusted RR	
	No (%)	Yes (%)	RR (95%CI^c^)	p-value	aRR (95%CI)	p-value	No (%)	Yes (%)	RR (95%CI)	p-value
**Total**	446 (84%)	87 (16%)					43 (49%)	44 (51%)		
**Public Health Service (PHS)**										
PHS 1	129 (86%)	21 (14%)	1				3 (14%)	18 (86%)	1	
PHS 2	65 (90%)	7 (10%)	0.7 (0.3–1.6)	0.38			5 (71%)	2 (29%)	0.7 (0.5–0.9)	0.01
PHS 3	152 (80%)	37 (20%)	1.4 (0.9–2.3)	0.18			24 (65%)	13 (35%)	0.7 (0.6–0.8)	0.00
PHS 4	39 (77%)	12 (23%)	1.7 (0.9–3.2)	0.11			6 (50%)	6 (50%)	0.8 (0.7–1.0)	0.04
PHS 5	61 (86%)	10 (14%)	1.0 (0.5–2.0)	0.99			5 (50%)	5 (50%)	0.8 (0.7–1.0)	0.06
**Gender**										
Female	265 (87%)	40 (13%)	1		1		19 (48%)	21 (52%)	1	
Male	181 (79%)	47 (21%)	1.6 (1.1–2.3)	0.02	1.5 (1.0–2.2)	0.06	24 (51%)	23 (49%)	1.0 (0.9–1.1)	0.74
**Age**										
0–24 years	125 (93%)	10 (7%)	1		1		3 (30%)	7 (70%)	1.3 (1.0–1.6)	0.03
25–34 years	230 (83%)	46 (17%)	2.3 (1.2–4.3)	0.02	2.5 (1.3–4.8)	0.01	20 (44%)	26 (56%)	1.2 (1.0–1.4)	0.07
≥ 35 years	91 (75%)	31 (25%)	3.4 (1.8–6.7)	0.00	3.5 (1.8–6.8)	0.00	20 (65%)	11 (35%)	1	
**TB incidence country of origin**										
50-99/100.000	126 (93%)	9 (7%)	1		1		5 (56%)	4 (44%)	1	
100-199/100.000	60 (77%)	18 (23%)	3.5 (1.6–7.3)	0.00	3.0 (1.4–6.6)	0.01	5 (28%)	13 (72%)	1.2 (0.9–1.5)	0.18
≥200/100.000										
*Other countries*	99 (80%)	24 (20%)	2.9 (1.4–6.1)	0.00	3.3 (1.6–6.7)	0.00	9 (37%)	15 (63%)	1.1 (0.9–1.5)	0.36
*India*	161 (82%)	36 (18%)	2.7 (1.4–5.5)	0.01	2.7 (1.3–5.3)	0.01	24 (67%)	12 (33%)	0.9 (0.7–1.2)	0.54
**Intended stay in the Netherlands**										
6 months, <5 years	274 (85%)	47 (15%)	1				29 (62%)	18 (38%)	1	
≥ 5 years	172 (81%)	40 (19%)	1.3 (0.9–1.9)	0.20			14 (35%)	26 (65%)	1.2 (1.0–1.4)	0.01
**Employment status**										
Employed	166 (82%)	37 (18%)	1				22 (60%)	15 (40%)	1	
Unemployed	158 (79%)	41 (21%)	1.1 (0.8–1.7)	0.55			17 (42%)	24 (58%)	1.1 (1.0–1.3)	0.11
School	122 (93%)	9 (7%)	0.4 (0.2–0.8)	0.01			4 (44%)	5 (56%)	1.1 (0.9–1.4)	0.40
**Highest completed education**										
Higher education	380 (85%)	67 (15%)	1		1		39 (58%)	28 (42%)	1	
No higher education	66 (77%)	20 (23%)	1.6 (1.0–2.4)	0.05	1.6 (0.9–2.6)	0.09	4 (20%)	16 (80%)	1.3 (1.1–1.4)	0.00

^a^ All case analyses

^b^ RR = Risk Ratio

^c^ CI: Confidence Interval

Of 94 clients diagnosed with LTBI, 49 (52%) initiated and 34 (69%) completed LTBI treatment. Treatment initiation proportions differed considerably between PHSs (29–86%) and declined with increasing age, from 7% among those 0–17 years to 27% among those 45 years and older. (Table 1) Clients were more inclined to initiate LTBI treatment when their intended stay in the Netherlands was ≥5 years, and when having lower levels of education. (Table 2) TB physicians reported contra-indications as a reason for eight (18%) of 45 clients not initiating LTBI treatment. Other common reasons reported for not initiating treatment were: no perceived advantages of LTBI treatment by client and return of the client to country of origin in the foreseeable future. The most common reason for discontinuation of treatment (31% of clients) was side-effects. Challenges encountered by TB physicians during client’s LTBI treatment are summarized in Table 3. TB physicians reported that they did not perceive language as a barrier when consulting most (81%) of their clients. In case of language barriers, usually a family member was used as an interpreter during consultations. Most (76%) clients who initiated LTBI treatment received support by the TB nurse. (Table 3)

**Table 3:** Evaluation of latent tuberculosis treatment by the physician.

	n	(%)
**Total clients diagnosed with LTBI**	**94**	**(100)**
**Language experienced as barrier during consultations?**		
No, not at all	76	(81)
Yes, a little	12	(13)
Missing	6	(6)
**Was a translator used during consultations?**		
No, it was not necessary	71	(76)
No, no translator was available	1	(1)
Yes, a family member performed as a translator	17	(18)
Missing	5	(5)
**Was contraindication a reason for not initiating LTBI treatment (n = 45)?**		
Yes ^a^	8	(18)
No	37	(82)
**Reasons other than contra-indications for not initiating LTBI treatment**^**b**^		
No perceived advantages of LTBI treatment by the client ^c^	16	(36)
Return of client to country of origin in foreseeable future	12	(27)
Objection against long duration of PT / afraid of side-effects	6	(13)
Dubious IGRA value	6	(13)
Unknown	5	(11)
**Total clients who initiated LTBI treatment**	**49**	(**52**)
**Reported side-effects during LTBI treatment**		
Yes	23	(47)
**Challenges experienced during LTBI treatment** ^**b**^		
Interruption of treatment	3	(6)
Difficulties with follow-up appointments	6	(12)
Difficulties with duration of LTBI treatment	2	(4)
No understanding of difference LTBI and active TB	1	(2)
False-diagnosis of MDR-TB	1	(2)
Problems within family: difficulty reaching the client for follow-up	1	(2)
**LTBI treatment support given by TB nurse**		
Yes	33	(67)
*< 10 times*	*31*	*(63)*
*≥ 10 times*	*2*	*(4)*
**Reason for discontinuing LTBI treatment (n = 15)**		
Side-effects ^d^	9	(18)
Pregnancy	2	(4)
Withdrawn for unknown reasons	4	(8)

^a^ Contra-indication reported: medication use (n = 2), end stage cancer (n = 1), pregnancy / wish to become pregnant in the foreseeable future (n = 2), psychosocial complaints (n = 2), missing (n = 1)

^b^ Multiple reasons / problems can be reported for one client

^c^ includes: low perceived chance of developing active TB, client does not understand utility of LTBI treatment

^d^ Side effects reported: hepatotoxicity (n = 2), other (n = 7)

### Enhancers and barriers for LTBI screening and treatment

Most interviewed PHS staff endorsed the importance of a future LTBI screening and treatment program among immigrants to reach a 25% decline in TB incidence in the Netherlands. However, PHS staff did experience some barriers for execution of the program.

Providing information about screening procedures is a legal obligation of PHS. Despite the available information brochures about the LTBI screening, some clients were still uninformed during the LTBI screening because a few PHSs (n = 2) did not send the information brochure prior to the LTBI screening as screening appointments were made by phone. Other clients had been misinformed about screening procedures through information channels (colleagues, companies, relatives) and therefore wrongly believed that the screening comprised a CXR. PHS staff said that clients who were not correctly informed about screening procedures were often more agitated because the LTBI screening was more time-intensive than the expected CXR. Another misunderstanding among some clients was that screening outcomes would affect their residence permit.

The health questionnaire is considered an essential part of the LTBI screening. However, we noted frequently that reported comorbidity and medication were inaccurate and was not well evaluated by the MTAs. Furthermore, and despite training, the importance of the questions was not sufficiently clear to all MTAs, which negatively influenced the quality of the evaluation of the answers and initiation of the required follow-up steps.

*MTA PHS1: “When you evaluate the health questionnaire and a client asks you why certain information is required, you notice that you need more in-depth understanding of underlying reasons for asking those questions*.*”*

We found suboptimal LTBI treatment initiation (52%). Some TB physicians were under the impression that they could influence the clients’ choice on initiation of LTBI treatment. This is reflected by the broad treatment initiation range (29–85%): the PHS with the “intention to screen is intention to treat” attitude achieved initiation proportion of 86%. TB physicians of the three PHSs with the lowest proportions (29–50%) were more skeptical about offering LTBI treatment to immigrants with a short-intended duration of stay in the Netherlands. This is reflected by the higher risk ratio for those with intended stay of ≥5 years (RR: 1.3 (95%CI: 1.1–1.4)). TB physicians argued that for immigrants with a short-intended stay and high risk for re-infection in the home country, the individual and public health benefits would not outweigh the risks of LTBI treatment.

*TB physician PHS4: “I am not in favor of initiating LTBI treatment when a client will return to Sub Saharan Africa because it is impossible for this client to live in Sub Saharan Africa without getting re-infected with TB*. *(….) However, for clients from northern Africa LTBI treatment might be favorable.”*

*TB physician PHS1: “For someone who will not even be here for one year the chance for that client to develop TB disease and therefore endanger our public health is low*. *Therefore, I would like to discuss duration of stay before we implement LTBI screening and treatment as national policy.”*

The guidelines states that, following the recommendations of the manufacturer, LTBI diagnosis is confirmed in clients with an IGRA result ≥0.35 in whom TB disease or history of TB disease is excluded. However, some TB physicians question this recommended cut-of-value, particularly among clients with a low reported risk of exposure. For this reason, six clients at one PHS were not offered LTBI treatment.

TB Physician PHS2: *“It [LTBI diagnosis] depends on exposure*, *recent or old infection*, *logistical factors*, *a lot of factors*. *So*, *IGRA results between 0*.*35 and 1*.*00 -the so-called grey area- can go either way*. *You can repeat the test*, *or you can just say*: *no*, *it is not an LTBI if anamnesis factors are not in favor*. *So*, *basing LTBI diagnosis solely on an IGRA result of 0*.*35 is not enough*.*”*

Quantitative results showed that clients with lower levels of education were more inclined (RR: 1.3 (95%CI: 1.1–1.4)) to initiate LTBI treatment than those with higher levels of education. TB physicians perceived clients with difficulties to understand the concept of LTBI and who confused their diagnosis with TB disease, and clients with fear for developing TB disease were more inclined to initiate LTBI treatment.

*TB Physician PHS3: “I believe that people from certain countries do not understand the difference between TB disease and LTBI and therefore want treatment*. *I can explain the difference a hundred times, but they just do not see it. (…) They say: ‘Oh, yes I have TB therefore I need treatment’.”*

PHS staff said that some higher educated clients could not believe they had been diagnosed with LTBI. These clients often perceived TB as a disease of the poor or thought TB disease did not occur in their home country anymore. PHS staff said those clients were less likely to initiate treatment.

*TB nurse PHS4: “They say: ‘There is no TB in our country!’*. *That is when I showed the TB incidence list of the World Health Organization and told them: ‘Sorry, but the World Health Organization disagrees with you’.”*

*MTA PHS5: “I can remember a client from India, who had a positive IGRA test result*. *The client said: ‘How is this even possible, this only occurs among the poor’.”*

Furthermore, PHS staff perceived unfamiliarity with LTBI treatment as a barrier for LTBI treatment initiation. Clients who did understand prevention and the benefits of LTBI treatment were more inclined to initiate LTBI treatment.

*MTA PHS4: “I remember this client in whom you [TB nurses] had invested a lot of time. She came to me and said: “I finally understand: he [the TB bacterium] is sleeping. So, he is present in the body but not active. So, you do not want to awake him*. *That is why you need to take medication”.*

Some PHS staff said that some client’s lack of trust in the Dutch TB control system complicated treatment initiation. They experienced that some clients, especially from India, China and Indonesia, consulted their physician in their home country about their LTBI diagnosis and treatment options. PHS staff perceived that those physicians were unfamiliar with purposes of LTBI screening and treatment activities in low TB incidence countries and therefore advised their clients against LTBI treatment.

*TB physician PHS4: “What I noticed, you do not win much trust from some clients*. *They always want to consult a physician in their own country. Especially clients from India. (….) they [physicians in home country] will tell what will eventually happen.’*

TB nurses said that the frequency and intensity of treatment support generally depends on level of education: clients with lower levels of education need more intensive treatment support. A barrier that some TB nurses experienced was that clients were hard to reach during office hours for phone appointments, which are used to monitor treatment progress.

*TB nurse PHS1: “Some clients were sometimes hard to reach by phone*. *We wanted to call to ask how they were doing. But because of work they did not answer our calls: they would call back at 11 at night. We need to draw a line somewhere…”*

PHS staff experienced the LTBI screening and treatment program as more time-intensive than CXR screening and some therefore questioned the cost-effectiveness. Some other PHS staff, however, noted that with LTBI screening as policy, cost and time would be saved from cancelled CXR follow-up screening among those with a negative IGRA test result. PHS staff noted some conditions, related to testing and treatment costs, if LTBI screening and treatment would become national policy in the future. Some PHS staff said that LTBI screening with only IGRA would be a more efficient pathway: the client only must visit the PHS once instead of twice, and the PHS would save time in extra consultations because of the high rate of positive TST. However, PHSs consider IGRAs to be expensive at this moment (prices in the Netherlands range from 50–104 euros for a single IGRA test compared to 27 euros for a single TST test). They expected with an increased demand for IGRAs, laboratories should be able to offer them cheaper. Additionally, TB physicians reported that the costs of LTBI treatment -which we provided for free during this study- would be a major barrier for clients to initiate treatment (roughly 180 euros for LTBI medication, excluding hepatotoxicity tests and CXRs).

*TB physician PHS1: “I am in favor of screening and treating clients at entry for LTBI*. *I think it is a key strategy towards elimination of TB in the Netherlands. (…) But, the strategy is not only of individual interest but also of public interest. Therefore, medication must be offered free of charge. That is, I think, the only way the strategy will be successful.”*

*TB physician PHS2: “A few clients had to -accidentally- pay for their medication at the pharmacy*. *They were shocked by the price. So, the financial compensation, I think, plays a key role in the success of the program.”*

## Discussion

We used mixed methods to study the implementation of LTBI (entry-)screening and treatment among newly-arriving immigrants in the Netherlands. We screened 566 immigrants, of whom three (0.5%) had TB disease, and 94 (17%) were diagnosed with LTBI. Forty-nine (52%) clients initiated and 34 (69%) completed LTBI treatment. Of clients screened with TST, 72% had a positive TST reaction which was considerably higher than the proportion (45%) found in a previous study. [10] The high increase can be attributed to the introduction of a different PPD concurrent with our study. (Mulder C. personal communication) We found suboptimal LTBI treatment initiation which was hampered by both client and TB physician related factors: short intended duration of stay, clients’ unfamiliarity with prevention and LTBI treatment, stigma, and consultation of physicians in the home country by clients. Although encountering practical barriers, most PHS staff perceived the LTBI screening and treatment among immigrants as an appropriate strategy to replace CXR screening, provided intended duration of stay in the Netherlands was long and both LTBI screening and treatment were offered free of charge; i.e. not deducted from the obligatory deductible excess (385 euros) for health insurance.

From this study, we learned that current information provision (information brochures and minor explanation about LTBI screening procedures) is insufficient. To avoid misinformation of clients, information about LTBI screening purposes and procedures should be more culturally sensitive and provided prior to the LTBI screening. Also, stakeholders and relevant organizations should be informed about changes in TB screening practices to ensure correct information sharing, such as between clients and employers. Furthermore, future information materials should emphasize more that LTBI screening outcomes do not influence obtainment of a residence permit. Additionally, future practices should consider engaging cultural mediators, who can overcome barriers related to culture and literacy, when working with a foreign-born population.[17] Using cultural mediators may improve adherence to the screening program, including initiation and completion of LTBI treatment. [18–21]

LTBI treatment initiation, adherence and completion determine both individual and public health benefits and the success of LTBI screening and treatment programs. [22] Although our LTBI treatment initiation proportion (52%) was within the range of initiation proportions among immigrants (23–97%) reported in other studies, it is low when compared to initiation of LTBI treatment among other target groups in the Netherlands (77%). [5, 23] These target groups, however, consisted mainly of TB contacts (85%), among whom treatment initiation is generally higher than among immigrants. [5, 7, 23] Despite having addressed barriers identified in a previous study, LTBI treatment initiation in the present study was only 5% higher than in the earlier one (52% versus 47%). [10] Other factors therefore seem to be equally important barriers for LTBI treatment initiation. We observed large differences in LTBI treatment initiation (varying from 28% to 86%) between PHSs: the TB physicians of one PHS with “intention to test is intention to treat” view achieved the highest proportion of 86%. Gutsfeld et al. also related low proportions of LTBI treatment initiation to the attitude of physicians who were not convinced of LTBI treatment benefits and efficacy. [24]

In our study, some TB physicians questioned individual and Dutch public health benefits of LTBI treatment among clients returning to high incidence countries within the foreseeable future. TB physicians perceive the risk for re-infection among immigrants returning to high incidence countries to be so high, that there is low individual benefit from LTBI treatment. However, Houben et al. found that only 1.5% and 1.2% of the population in WHO African Region and the WHO Southeast Asia Region respectively was recently infected (last two years). [25] TB physicians also said that the Dutch public health benefit would be small as chances of development of TB disease among immigrants in the period after arrival would be low. However, new immigrants do contribute to TB incidence in the Netherlands: 75 (8%) immigrant TB patients in 2016 stayed less than 2.5 years in the Netherlands. [7] Furthermore, clients who do not initiate LTBI treatment, are educated on symptoms suggestive of TB disease and the need to seek medical attention when such symptoms present themselves. Consequently, they are likely to have shorter diagnostic delays. We thus argue that both the individual and public health benefit of LTBI screening of immigrants with a relative short intended stay in the Netherlands may be underestimated.

TB physicians noted that clients who had less understanding of the difference between LTBI and TB disease, and clients who had more fear of TB were more inclined to initiate LTBI treatment. In our study we noted that clients with lower levels of education were more inclined to initiate LTBI treatment. Goswami et al. showed that lower levels of education and fear of getting TB disease were independently associated with LTBI treatment initiation. [26] PHS staff said that some higher educated clients considered TB as a disease of the poor. This might be caused by or lead to stigma, which can lead to denial of diagnosis of TB disease and consequently complicate treatment initiation. [27] This might be the same for LTBI treatment initiation. Furthermore, clients consulting physicians in their home country also impeded treatment initiation: those physicians lacked awareness about the benefits of LTBI treatment and were likely not aware of the recommended policy to screen and treat new immigrants in low incidence countries for LTBI. It is therefore important to create more awareness and knowledge about TB and LTBI among both clients and healthcare workers in the home countries. [28]

In our study, 31% of clients did not complete LTBI treatment, which is suboptimal compared to completion proportions among other high-risk groups (overall 82%) in the Netherlands.[5] However, LTBI treatment completion in our study is in the higher range compared to the range among immigrants found by Sandgren et al. (proportions between 7–86%). [23] Unfortunately, due to small numbers we were not able to assess factors impeding completion of LTBI treatment.

Current modelling and cost effectiveness analysis do not differentiate treatment initiation and completion rates among varying migrant groups. [29] However, factors influencing LTBI treatment initiation and completion likely differ between and within different migrant groups such as immigrants and asylum seekers. Our operational study provides a unique insight into the challenges faced when optimizing the cascade of care of LTBI screening and treatment among immigrants. Our second pilot study will elaborate more on optimizing the cascade of care for LTBI screening and treatment among asylum seekers (Spruijt et al., in progress). Eventually mathematical and cost effectiveness models will be employed to evaluate optimized definitions of target groups within migrant populations regarding cost effectiveness and impact of LTBI screening and treatment programs in the Netherlands. Those models will generate an evidence for recommendations on specific target groups and an optimized LTBI screening and treatment program, that may also be applicable in other high income, low TB incidence countries.

This study has several limitations. First, as participation in the screening was mandatory we could not calculate acceptation rates of LTBI screening. This would have been useful to determine the feasibility of voluntary screening programs. Secondly, many international companies reside in participating PHSs regions. Therefore, the non-labor immigrant population may be underrepresented in our study. Furthermore, we did not screen foreign students for practical reasons. Despite potential underrepresentation of non-labor immigrants and foreign students, we could not find considerable differences in country of origin between the study population and the total immigrant population screened nationwide in 2016. [6, 7] Thirdly, we could not assess independent factors associated with treatment initiation or completion because the number of clients initiating LTBI treatment was too small. A multivariable analysis would consequently have too many degrees of freedom, which may lead to overfitting of the data and induce sparse data bias. Consequently, we would obtain estimates of the regression coefficients which are not replicable in future samples and do not represent the true effect.[30, 31] However, combining both quantitative and qualitative results does give a good impression of existing barriers for LTBI treatment initiation and completion.

## Conclusion

In a mandatory LTBI screening program among new immigrants from high endemic countries, LTBI treatment initiation did not increase considerably after removing costs: misconceptions and doubts about the benefits and effectiveness of the intervention among both clients and providers hampered LTBI treatment initiation. Adoption of an “intention to test is intention to treat” view by TB physicians could increase treatment initiation considerably. Additionally, cultural mediators and culturally sensitive education tools should be used to overcome barriers for immigrants related to their understanding of LTBI and the consequent cascade of care. Future modelling and studying the cost-effectiveness of different screening scenarios will contribute to better targeting migrant subgroups that will benefit most from LTBI screening and treatment interventions and optimize the impact of TB prevention efforts on the occurrence of TB in the country.

## Supporting information

S1 Fig Flowchart of Latent tuberculosis infection screening process. (PDF)

S1 Table Levesque’s conceptual framework and interview topic guide. (PDF)
