# Supplementary material for: Implementation of latent tuberculosis infection screening and treatment among newly arriving immigrants in the Netherlands: A mixed methods pilot evaluation
Source: PLoS One. 2019 Jul 1;14(7):e0219252. doi: 10.1371/journal.pone.0219252 (PMC6602457; doi:10.1371/journal.pone.0219252)
Supplement: S1 Fig — (PDF) [file pone.0219252.s001.pdf]

S1 Fig. Flowchart of Latent tuberculosis infection screening process.

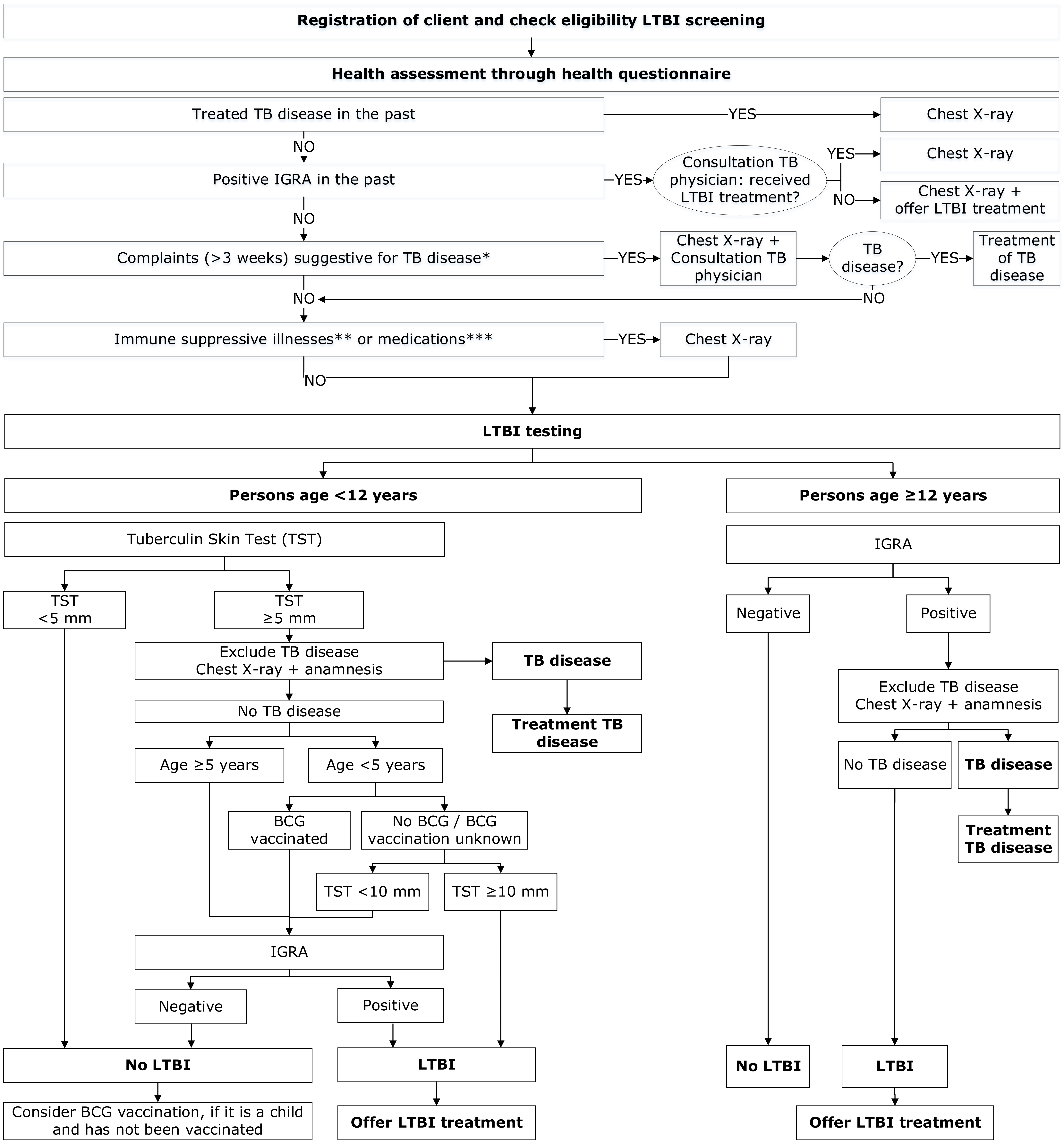

*Complaints: Cough, fever (>38 degrees Celsius), night sweats, poor appetite / weight loss
**Immune suppressive illnesses: Inflammatory bowel disease, kidney failure / dialysis, diabetes, HIV/AIDS, cancer, organ transplantation, psoriasis, rheumatism, sarcoidosis, silicosis
*** Immune suppressive medications: Prednisone / Dexamethasone / Methotrexate, TNF-alpha blockers (biologicals), cancer medication, medication following organ transplantation

Acronyms: TB: tuberculosis, LTBI: latent tuberculosis infection, IGRA: Interferon Gamma Release Assay, BCG: Bacillus Calmette-Guérin
