# Supplementary material for: Implementation of latent tuberculosis infection screening and treatment among newly arriving immigrants in the Netherlands: A mixed methods pilot evaluation
Source: PLoS One. 2019 Jul 1;14(7):e0219252. doi: 10.1371/journal.pone.0219252 (PMC6602457; doi:10.1371/journal.pone.0219252)
Supplement: S1 Table — (PDF) [file pone.0219252.s002.pdf]

S1 Table. Levesque's conceptual framework and interview topic guide.

Levesque's five dimensions for access to care*	Explanation of the dimension	Primary questions in topic guide
Approachability	People facing health needs can actually identify that some form of services exists, can be reached, and have an impact on the health of the individual	**Information about the LTBI screening**<br>What information about the screening is currently available, and what are unmet needs?
		**Understanding LTBI diagnosis**<br>How did clients perceive LTBI diagnosis and what factors played a role in understanding the LTBI diagnosis?
Acceptability	Cultural and social factors determining the possibility for people to accept the aspects of the service and the judged appropriateness for the persons to seek care.	**Acceptability of LTBI screening**<br>How do you think immigrants experience the LTBI screening, and what factors enhance or impede acceptability?
		**Provision and acceptability of LTBI treatment**<br>How is LTBI treatment provided, and what factors enhance or impede acceptability of LTBI treatment by clients?
Availability and accommodation	Health services (either the physical space or those working in health care roles) can be reached both physically and in a timely manner.	**Resources required for LTBI screening**<br>What resources were required for the LTBI screening?
		**Resources required for LTBI treatment**<br>What resources were required for the LTBI treatment?
Affordability	The economic capacity for people to spend resources and time to use appropriate services.	**Financial needs for LTBI screening**<br>What human and financial resources play a role in the LTBI screening?
		**Financial needs for LTBI treatment**<br>What human and financial resources play a role in the LTBI treatment?
Appropriateness	The fit between services and clients need, its timeliness, the amount of care spent in assessing health problems and determining the correct treatment and the technical and interpersonal quality of the services provided.	**Execution of LTBI screening**<br>What were enhancers and barriers for executing LTBI screening and for clients' satisfaction?
		**Execution of LTBI screening**<br>What were enhancers and barriers for the initiation, continuation and completion of LTBI treatment?

*Levesque JF, Harris MF, Russell G. Patient-centred access to health care: conceptualising access at the interface of health systems and populations. Int J Equity Health. 2013;12:18
